# Incidence of "quasi-ditags" in catalogs generated by Serial Analysis of Gene Expression (SAGE)

**DOI:** 10.1186/1471-2105-5-152

**Published:** 2004-10-18

**Authors:** Sergey V Anisimov, Alexei A Sharov

**Affiliations:** 1 Section for Neuronal Survival, Wallenberg Neuroscience Center, Lund University, 221 84 Lund, Sweden; 2 Laboratory of Genetics, National Institute on Aging, National Institutes of Health, Baltimore, MD, 21224, USA

## Abstract

**Background:**

Serial Analysis of Gene Expression (SAGE) is a functional genomic technique that quantitatively analyzes the cellular transcriptome. The analysis of SAGE libraries relies on the identification of ditags from sequencing files; however, the software used to examine SAGE libraries cannot distinguish between authentic versus false ditags ("quasi-ditags").

**Results:**

We provide examples of quasi-ditags that originate from cloning and sequencing artifacts (i.e. genomic contamination or random combinations of nucleotides) that are included in SAGE libraries. We have employed a mathematical model to predict the frequency of quasi-ditags in random nucleotide sequences, and our data show that clones containing less than or equal to 2 ditags (which include chromosomal cloning artifacts) should be excluded from the analysis of SAGE catalogs.

**Conclusions:**

Cloning and sequencing artifacts contaminating SAGE libraries could be eliminated using simple pre-screening procedure to increase the reliability of the data.

## Background

Serial Analysis of Gene Expression (SAGE) is a rapid method to study mRNA transcripts in cell populations [1]. Two major principles underline SAGE: (1) short expressed sequenced tags (ESTs) are sufficient to identify individual gene products, and (2) multiple tags can be concatenated and identified by sequence analysis [1,2]. With the ever-expanding sequence information available in public databases, identification of gene transcripts with SAGE tags has greatly facilitated transcriptome comparisons and gene identification [3].

SAGE data are usually analyzed with software packages like "SAGE300" or "SAGE2000". The majority of SAGE libraries use *NlaIII *or *Sau3A *(*SalAI*) as anchoring enzymes (AE) to create SAGE tags. Both of these enzymes have 4-bp palindromic recognition sequences (CATG for *NlaIII *and GATC for *Sau3A*) that flank individual ditags within concatemers. A major component of the software analysis is the identification of anchoring enzyme recognition sequences (AERS) that flank target sequences (SAGE ditags). After finding the first AE recognition sequence, the software continues reading the sequence until it finds the next one. The software then compares the distance between these recognition sequences with predicted ditag lengths (20–24 bp in the case of *NlaIII *or *Sau3A*), and ditags that are too short (<20 bp) or too long (>24 bp) are excluded. However, if the length of the AERS-flanked sequence satisfies the size criteria, it is identified as a ditag. This algorithm relies on the assumption that *all *sequences have correctly organized ditag concatemers; however, the cloning efficiency of SAGE rarely reaches 100%. In this report, we show that up to 5% of ditags from some SAGE libraries should be omitted from the final analysis. These false ditags (termed "quasi-ditags") result from genomic contaminants and apparently random combinations of nucleotides generated by cloning or sequencing errors. Using a mathematical model to simulate the frequency of quasi-ditags in DNA, we propose a method to exclude quasi-ditags from SAGE catalogs.

## Results

**Figure 1:**
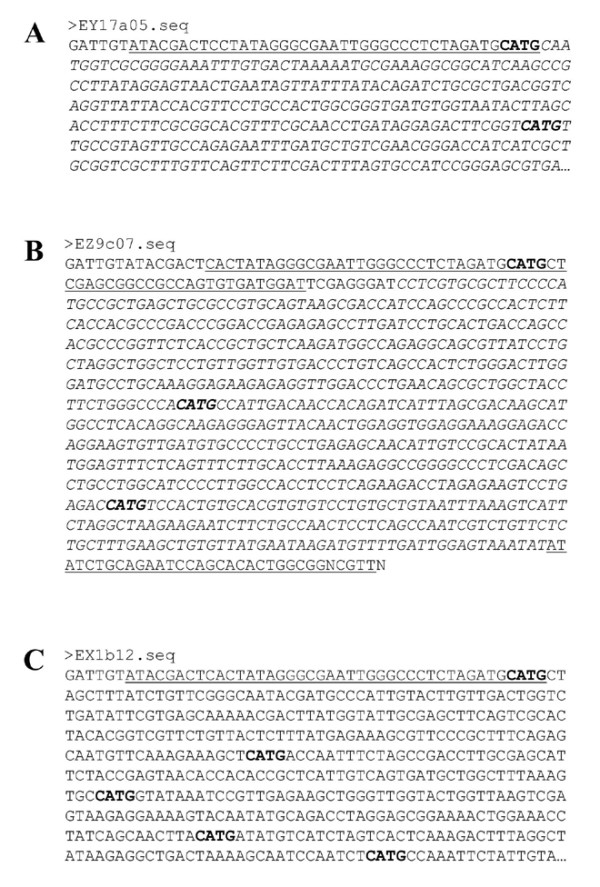
Raw SAGE sequence data showing cloning and potential sequencing artifacts excluded by SAGE software. (A) Clone with fragment of *E. Coli *genomic DNA. Italics denote *E. Coli *sequence (AE000256). (B) Clone containing a fragment of rodent genomic DNA. Italics denote *M. musculus *sequence (AI894042). (C) Clone with unidentifiable insert, which lack normal SAGE concatemer. pZErO-1 sequences are underlined; Anchoring enzyme recognition sites (AERS_(CATG)_) are shown in bold.

From twelve independent SAGE libraries, we analyzed numerous clones lacking organized ditag concatemers that would be excluded by SAGE software packages, including clones lacking inserts, clones with inserts containing bacterial or rodent genomic sequences, and clones with unidentifiable sequences (Figure 1). Depending on the quality of the SAGE library, examples of clones in Figure 1A,1B,1C can represent up to 50% of the total volume of clones sequenced [4], but generally range from 2–20%. A more typical example is taken from our R1 ES cell and AMH-II SAGE libraries [5,6], which contained 5,988 and 4,478 clones, respectively. The cloning efficiency was ~79% and ~76% (4,714 and 3,413 clones with inserts, respectively). Among these, 411 and 167 clones in the R1 ES SAGE library and 305 and 194 clones in the AMH-II library contained sequences with only 1 or 2 ditags, respectively.

**Figure 2:**
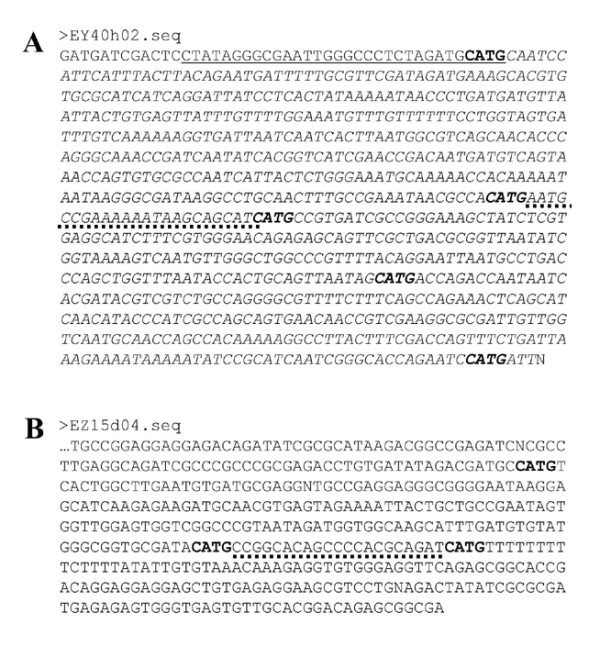
Raw SAGE sequence data showing cloning and potential sequencing artifacts not excluded by SAGE software. (A) Clone with fragment of *E. Coli *genomic DNA. Italics denote *E. Coli *sequence (AE000307). (B) Clone with unidentifiable insert, which lack normal SAGE concatemer. Sequences like (A-B) represent quasi-ditags that should have been removed. pZErO-1 sequence is underlined; Anchoring enzyme recognition sites (AERS_(CATG)_) are shown in bold, and potential SAGE tags are shown by dotted underlines.

During our sequence analysis of the clones that had produced a least number of ditags (1–2 per clone), we identified a subset of sequences (up to 40%) that contain ditags that may be false. Importantly, some of these "ditags" matched bacterial genomic sequences (Figure 2A), while others seemed to represent random combinations of nucleotides. Figure 2B show an example of a clone that contains a single ditag sequence embedded within a sequence of unidentifiable origin. Because most of this sequence is not composed of concatenated ditags, this embedded ditag may therefore represent a quasi-ditag, which should be excluded from further analysis. These two examples, among others, suggest that some inserts in pZErO-1 contain sequences that just by chance mimic SAGE ditags.

**Figure 3:**
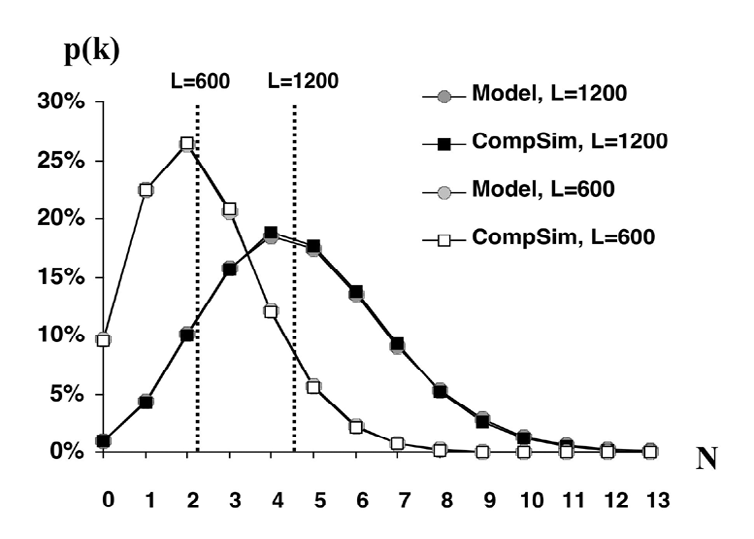
Probability (p(k)) to find k AERS_(CATG) _in a random sequence for L = 600 and L = 1200 bp. Dotted lines represent p(k) mean values. L, sequence length; Model, mathematical modeling; CompSim, computer simulation (1,000,000 simulations).

**Figure 4:**
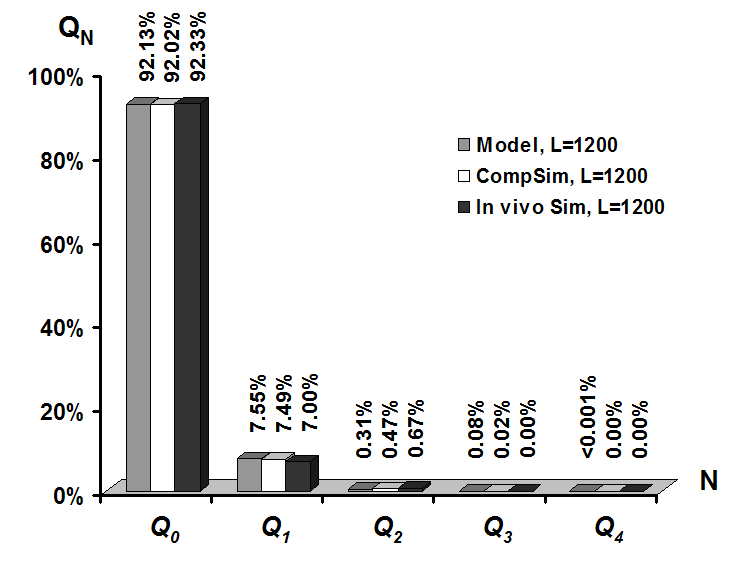
Probability to find various numbers of quasi-ditags (*Q*_*N*_) in the same nucleotide sequence of the given length (L = 1200 bp). L, sequence length; Model, mathematical modeling; CompSim, computer simulation (1,000,000 simulations); In vivo Sim, fragments of *S. Cerevisiae *chromosome (300 simulations).

**Figure 5:**
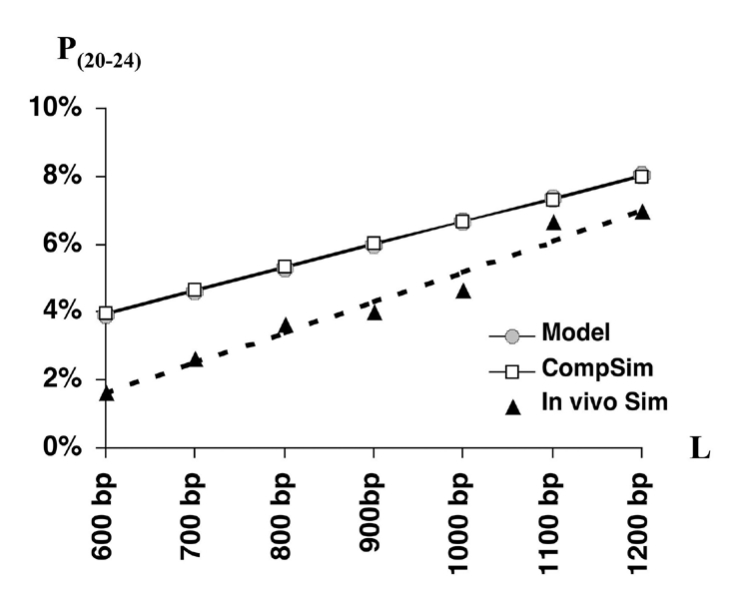
Probability of finding one or more quasi-ditag in the nucleotide sequence of a given length (L = 600 to 1200 bp). Model, mathematical modeling; CompSim, computer simulation (1,000,000 simulations for each L value); In vivo Sim, fragments of *S. Cerevisiae *chromosomes (300 simulations for each L value). Dotted line represents trendline for In vivo Sim.

To predict the potential frequency of randomly occurring quasi-ditags, we employed a stochastic model system to generate random sequences. We then used both computer-generated random sequences and true genomic DNA sequences to test this possibility. Random sequences were generated and analyzed with a Visual Basic program designed to mimic SAGE software analysis of ditags. The simulated sequences varied in length from 600 to 1200 nucleotides, which corresponds to the average sequence lengths generated by automated sequence analyzers. One million random sequence strings with L = 600, 700, 800, 900, 1000, 1100, and 1200 nucleotides were generated. Table 1 shows expected frequencies of quasi-ditags according to the model (equation (5)) and the observed frequencies based on computer simulations. The line plots of the expected (model) and observed (computer simulation) quasi-ditag frequencies are almost identical (Figures 3 and 4). Fragmented *Saccharomyces cerevisiae *genomic DNA that lack SAGE ditag concatemers was also employed for *in vivo / in silico *model validation, and a number of quasi-ditags was detected in these (Figures 4 and 5). When compared to *Saccharomyces cerevisiae *genomic DNA [7], quasi-ditag frequencies were somewhat less abundant than those generated by the computer, potentially due to the presence of nucleotide repeats and unequal frequencies of individual nucleotides in the Yeast genomic sequences. These data, however, support our hypothesis that quasi-ditags can be generated randomly from potential sequencing errors or from genomic contaminants. This analysis furthermore underscores the limited extent that quasi-ditags occur: the distribution of expected number of quasi-ditags per clone is clearly bimodal, with peaks at 1 and 2 ditags (*Q_1 _*and *Q_2_*, respectively). At the same time, the frequency of occurrence for three quasi-ditags (*Q_3_*) is extremely low (0.01% for L = 600 to 0.02% for L = 1200), such that the value of P^3 ^_(20–24) _effectively converges to zero for the majority of the SAGE catalogs (i.e. <3000–5000 clones) (Figure 4). Accordingly, the clones that include ditag concatemers of higher length should lack quasi-ditags.

**Figure 6:**
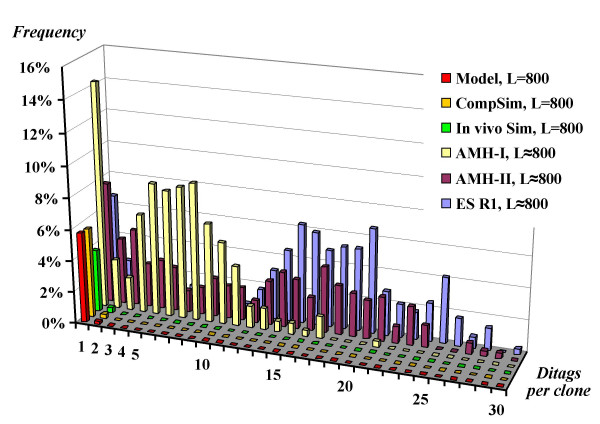
Frequency distribution of the number of ditags in SAGE output. Probability to find various numbers of ditags in the clone sequence has been plotted as a function of a number of total ditags per clone. Model, mathematical modeling; CompSim, computer simulation (1,000,000 simulations); In vivo Sim, fragments of *S. Cerevisiae *chromosome (300 simulations); AMH-I, -II, ES R1, actual SAGE data (sequences from 3 randomly chosen 96-well plates). Sequence length (L) = 800 bp for Model, CompSim and In vivo Sim; average sequence length ≈ 800 bp for all three SAGE libraries.

Clones containing only one or two ditags/quasi-ditags, however, could be excluded from SAGE analyses, without adversely affecting the data set (Figure 6). As an example, we extracted sequences from clones that produce 1–2 total ditags from AMH-II and R1 ES cell libraries. This reduced the total number of tags by 1.06% for ES R1 and 1.94% for AMH-II, but it effectively removed all contaminating bacterial sequences and improved the data reliability. However, the total AMH-I library (2,365 clones, ~78% cloning efficiency; [6]) had a larger proportion of ditags extracted as being too long (>24 bp), as indicated by lower tag per clone ratio (average insert size of 12.2 tags/clone vs. 22.6 in AMH-II library) amid the same average sequence length, suggesting higher proportion of quasi-ditags. Analysis of the AMH-I SAGE library has revealed 353 and 52 clones that contained just 1 or 2 ditags, respectively. Exclusion of these sequences decreased the total number of tags by 5.21% (calculated after duplicate dimer exclusion), and proved critical to our subsequent quantitative SAGE comparisons [6]. Failure to remove these quasi-ditag sequences decreased the quantitative reproducibility (R values) between AMH-I and AMH-II SAGE libraries, showing that quasi-ditags can adversely affect the reliability of SAGE libraries.

## Discussion

SAGE is an important tool of modern molecular biology widely used in a number of applications. We hypothesized that actual SAGE catalogs could be contaminated by false ditags ("quasi-ditags") of various origins. Although SAGE software packages are designed to ignore sequences that lack 20–24 bp sequences flanked by two anchoring enzyme recognition sites, it does not exclude quasi-ditags originating from genomic contaminants or unknown sequences that may arise as cloning or sequencing artifacts (Figure 2). Negative controls (self-ligated vector) do not produce any colonies after Zeocin selection and cannot account for the appearance of background clones and quasi-ditags in Zeocin-resistant bacteria. Since some quasi-ditags, however, originate directly from *E. Coli*, we suggest that one probable source for these contaminanting tags is from recombination events that occur in *E. Coli*. Indeed, such a mechanism has already been documented [8] and has led to the development of Stbl2 bacteria that are mcrA^-^/mcrBC^-^hsdRMS^-^mrr^-^. Since pZErO-1 was not translated into recombination deficient bacteria (DH10B), large-scale amplifications of this plasmid within bacteria would be expected to lead to some random recombinations, and the generation of quasi-ditags (e.g. Figure 2A).

Some of the ditags derived from the clones that had produced a least number of ditags (1–2 per clone) do not match genomic sequences and thus might be originated from sequencing errors. We therefore suggested a model that provides a mathematical basis for the hypothesis that such a possibility exists. The mathematical model presented in the manuscript is an attempt to predict the frequency distribution of quasi-ditags in random sequences. The phenomenon itself is rather complex and there is no simple model that would capture it in full complexity. We, however, believe that we have selected a reasonable level of model complexity that captures the major pattern of frequency distribution.

Using the computer simulation we show that random combinations of nucleotides generated could be indeed recognized by SAGE software as valid SAGE ditags. We also demonstrate that quasi-ditags may constitute a non-negligible proportion of SAGE catalogs. Our model, which simulates the frequency of quasi-ditags in DNA (equations (1–6)), suggests that single or double ditags may represent quasi-ditags; however, the results of the *in silico *experiments show that the probability of finding more than two quasi-ditags in the same sequence converges effectively to zero (Table 1 and Figure 4). Based on these findings, we suggest that additional steps be performed with SAGE libraries. We recommend removing clones with sequences containing ≤ 2 ditags at a pre-processing step ("clean-up"). The removal of clones containing 1 or 2 ditags can effectively remove bacterial genomic sequences and potential sequencing artifacts from SAGE libraries. The overall number of SAGE tags excluded by this additional step (authentic and quasi-ditags) is usually low, and generally does not exceed 1.0–1.8% of the total number of sequenced SAGE tags [5,6,9,10]; however, the frequency of potential quasi-ditags could be high (>5%) in some SAGE libraries. In AMH-I library, for example, the fraction of clones lacking appropriate ditag concatemers was >20%. In these instances, quasi-ditags significantly contribute to the final SAGE tag count, and should be removed.

Chart in Figure 6 plots values for ditag distribution from both the model-based simulations (L = 800 bp) and actual clones from the SAGE libraries that had sequences of the same mean length (L ≈ 800 bp). The expected maximum frequency of 1–2 quasi-ditags in the plotted model data approximated the observed frequency of clones with 1–2 total ditags detected in the pool of the actual SAGE clones. Contrary to that, the frequency of occurrence of three or more quasi-ditags predicted by the model is extremely low, demonstrating a divergence in the distribution of expected quasi-ditags and valid SAGE ditags for higher number of ditags per clone. Note that owing to the gel-purification of concatemers the majority of clones in the representative samples belong to the clusters of higher ditag numbers (AMH-II and ES R1 libraries, 13–26 total ditags; AMH-I library, 4–11 total ditags).

Comparing values of observed frequencies of the actual SAGE clones that produce 1–2 total ditags with those of expected quasi-ditag frequencies for the sequences of given length might be indicative on the possible contribution of cloning and sequencing artifact-derived quasi-ditags (Figure 6). The possible contribution of quasi-ditags to the final tag yield in SAGE libraries cannot be accurately predicted in advance but a failure to report the cloning efficiency and the number of clones with 1 or 2 ditags precludes an evaluation of potential false tags present in SAGE catalogs. Current SAGE protocols do not ensure 100% accurate size fractioning of concatemers: some of the smallest concatemers could therefore be cloned and sequenced. We recognize that some authentic tags (representing valid, but extremely short inserts that were not extracted during gel-purification of concatemers) will be excluded by removing all clones containing only 1 or 2 ditags. Nevertheless, we suggest that any potential loss of authentic ditags in the clean-up procedure is negligible compared to the advantage of having more reliable SAGE results.

SAGE protocols are extremely complex technologically and every possible mean should be employed to ensure qualitative and quantitative accuracy of catalogs on both the experimental and analytical steps. Evaluation of the cloning efficiency and precision (e.g. with RAST-PCR [11]) and sequencing accuracy are therefore essential on the stage preceding large-scale sequencing of the clones. Nonetheless, introduction of the simple pre-processing step eliminating false ditags would further improve the accuracy of the method resulting in its wider application.

## Conclusions

We have hypothesized that actual SAGE catalogs could be contaminated by false ditags (termed "quasi-ditags") of various origins and employed a mathematical model to predict the frequency of quasi-ditags in random nucleotide sequences. Cloning and sequencing artifacts contaminating SAGE libraries could be eliminated using simple pre-screening procedure to increase the reliability of the data.

## Methods

### SAGE

Serial analysis of gene expression (SAGE) was performed according to the original protocol [1] with minor modifications [5,12]. Human (PC3) and mouse (P19, R1, D3, EG-1, MEF) cells and tissues (adult and old heart) have been employed for construction of SAGE libraries and sequence analysis to illustrate the "clean-up" process. SAGE tags were generated with *NlaIII *and *BsmFI *restriction enzymes (New England Biolabs, Beverly, MA, USA). Sequencing was performed by Perkin-Elmer Applied Biosystems / Celera Genomics (Foster City, CA, USA) and Agencourt Bioscience Corporation (Beverly, MA, USA).

### Stochastic model

Anchoring enzyme recognition sites (AERS) are 4 bp long. Assuming for simplicity that all 4 nucleotide bases (A, T, C, and G) have equal frequencies, a probability that a random combination of 4 nucleotides would match the AERS is 4^-4 ^= 1/256. In a sequence of length *L*, the expected number of AERS (e.g. CATG for *NlaIII *anchoring enzyme) is *L*/256. Thus, the probability of finding *k *tags CATG in a random sequence of length L is determined by the Poisson distribution:


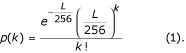


If two CATG sequences (AERS_(CATG)_) are located within the sequence of length *L*, then the probability that they are separated by a 20–24 bp distance (*P*_(20–24)_) is approximately:


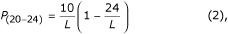


where 10 is the number of possible relative positions of two AERS_(CATG) _that yield a quasi-ditag and 24 is the mean distance from the center of one SAGE tag to the end of the sequence that does not leave enough space for another tag to form a quasi-ditag.

If >2 AERS are present in the sequence, then there is a chance that additional AERS would appear within the quasi-ditag formed by first two AERS. A probability that additional AERS will not appear within the quasi-ditag is approximately:


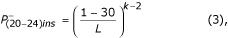


where 30 is the average length of a nucleotide string outside of the ditag.

If the total number of AERS_(CATG) _equals k, then the number of possible AERS pairs is:


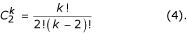


Taken together, a probability of at least one quasi-ditag in the sequence that has exactly *k *AERS_(CATG) _is:


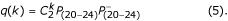


Then, a probability (*Q*_1_) to find at least one quasi-ditag in a sequence of given length *L *is:


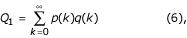


where p(k) is given by equation (1).

There is also a probability that more than one quasi-ditag exists within the sequence. In some cases the same AERS_(CATG) _could serve as a portion of the two neighboring quasi-ditags (...CATG-(N)_20–24_-CATG-(N)_20–24_-CATG...). In other cases, two or more quasi-ditags can be located independently in the sequence. If a sequence with *k *tags already has one quasi-ditag bounded by two tags, then other (k-2) tags may form additional quasi-ditags. The probability of existence of additional quasi-ditags on condition that one ditag is already present is approximately q(k-2). Then the total probability that any random sequence has at least two quasi-ditags is:


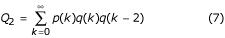


In the same way,


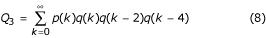


and so on.

The probability that a random sequence has exactly n quasi-ditags is:

*R*_*n *_= *Q*(*n*) - *Q*(*n *+ 1)     (9).

### Software and analysis

A random nucleotide generator (for L = 600–1200) and analysis program that mimics "SAGE300" or "SAGE2000" software algorithms was written in Visual Basic and is available upon request. Genomic DNA sequences of *Saccharomyces cerevisiae *that lack SAGE ditag concatemers were also employed for *in vivo / in silico *model validation. Randomly selected *S. cerevisiae *chromosomes were downloaded from GenBank, fragmented to create a minimum of 300 sequences (L = 600–1200) and searched for quasi-ditags using "SAGE2000" software (available at SAGE website [13]). Frequency distribution of the number of ditags was analyzed in raw sequences from 3 randomly chosen 96-well plates from AMH-I, AMH-II and ES R1 SAGE libraries (285 sequences for each library) using the same software.

## Authors' contributions

SVA developed the hypothesis, overall plan and performed SAGE, computer simulations, and analysis of Yeast genome fragments. AAS developed and implemented the mathematical model predicting the appearance of "quasi-ditags" in random sequences of given length. Both authors have contributed to the writing and approved the final manuscript.

## Figures and Tables

**Table 1:** Probability to find one or more "quasi-ditag" in the nucleotide sequence of the given length (P_(20–24)_)

Sequence Length (L)	Frequency of ≥ 1 quasi-ditags in sequence			*S. Cerevisiae chromosome *^3^
		
	Mathematical model	Computer simulation ^1^	In vivo ^2 ^simulation (S. Cerevisiae)	
600 bp	0.039392	0.039858	0.016666	IV [NC_001136]
700 bp	0.046231	0.046655	0.026666	X [NC_001142]
800 bp	0.053070	0.053383	0.036666	XIV [NC_001146]
900 bp	0.059909	0.060051	0.040000	VIII [NC_001140]
1,000 bp	0.066748	0.066743	0.046666	V [NC_001137]
1,100 bp	0.073587	0.073225	0.066666	IX [NC_001141]
1,200 bp	0.080426	0.079793	0.070000	XI [NC_001143]

^1 ^For computer simulation, 1,000,000 files consisting of the sequence-imitating random combination of A, C, G and T nucleotides of selected length were analyzed in search of SAGE "quasi-ditags".

^2 ^For *in vivo / in silico *simulations 300 sequences were created by fragmentation of randomly selected chromosomes of *Saccharomyces Cerevisiae *for each L value. Larger samplings (900–1,400 sequences) were created and tested for selected sequence lengths and did not change results significantly.

^3 ^GenBank database accession numbers are given in brackets.
